# Supplementary material for: Complexome profiling of the Chlamydomonas psb28 mutant reveals TEF5 as an early PSII assembly factor
Source: Plant Cell. 2025 Mar 17;37(6):koaf055. doi: 10.1093/plcell/koaf055 (PMC12164590; doi:10.1093/plcell/koaf055)
Supplement: koaf055_Supplementary_Data [file koaf055_supplementary_data.zip › psb28_interactive.zip!/plots/intensity/qualityPlots/peptides/CPLD33_Cre03.g145207.t1.1.html]

### Cre03.g145207.t1.1

Trivial name: CPLD33  
SeqCoverage [median]: 15.50  
Mass: 25.19 kDa  
  
 

| Sequence | MissedCleavage | Mass [Da] | wtIntensity | muIntensity | Score |
| --- | --- | --- | --- | --- | --- |
| QIAGTFAPR | 0 | 959.52 | 1119.44 | 1600.68 | 83.21 |
| SGNEQQTTTEQPSTSSTPSVEKDILR | 1 | 2819.34 | 0.00 | 2964.65 | 86.21 |
|  |  |  | 1119.4 | 4565.3 |  |
